# The Importance of Selected Dysregulated microRNAs in Diagnosis and Prognosis of Childhood B-Cell Precursor Acute Lymphoblastic Leukemia

**DOI:** 10.3390/cancers15020428

**Published:** 2023-01-09

**Authors:** Karolina Joanna Ziętara, Jan Lejman, Katarzyna Wojciechowska, Monika Lejman

**Affiliations:** 1 Student Scientific Society, Laboratory of Genetic Diagnostics, Medical University of Lublin, 20-093 Lublin, Poland; 2 Independent Public Health Care Facility of The Ministry of Internal Affairs and Administration in Lublin, 20-331 Lublin, Poland; 3 Independent Laboratory of Genetic Diagnostics, Medical University of Lublin, 20-059 Lublin, Poland

**Keywords:** childhood, B-cell precursor acute lymphoblastic leukemia, miRNA, diagnosis, prognosis, biomarker

## Abstract

**Simple Summary:**

B-cell precursor acute lymphoblastic leukemia (BCP-ALL) is one of the most common hematological malignancies of childhood. The precursors present in the bone marrow give rise to cells that then begin to multiply uncontrollably. Molecules such as microRNA (miRNA) take part in BCP-ALL oncogenesis. They are short, non-coding, single-stranded RNAs with the regulatory potential of gene expression. miRNA has an influence on both pre- and post-translational processes. The stability of form and the presence of miRNA in circulation are useful in the diagnostic and prognostic processes of pediatric leukemias. miRNAs are gaining the interest of scientists due to their biomarker potential and easy determination.

**Abstract:**

B-cell precursor acute lymphoblastic leukemia (BCP-ALL) is a frequent type of childhood hematological malignancy. The disease is classified into several subtypes according to genetic abnormalities. MicroRNAs (miRNAs) are involved in pathological processes (e.g., proliferation, apoptosis, differentiation). A miRNA is a group of short non-coding RNAs with relevant regulatory effects on gene expression achieved by suppression of the translation or degradation of messenger RNA (mRNA). These molecules act as tumor suppressors and/or oncogenes in the pathogenesis of pediatric leukemias. The characteristic features of miRNAs are their stable form and the possibility of secretion to the circulatory system. The role of miRNA in BCP-ALL pathogenesis is still emerging, but several studies have suggested using miRNA expression profiles as biomarkers for diagnosis, prognosis, and response to therapy in leukemia. The dysregulation of some miRNAs involved in childhood acute lymphoid leukemia, such as miR-155, miR-200c, miR-100, miR-181a, miR125b, and miR146a is discussed, showing their possible employment as therapeutic targets. In the current review, the capabilities of miRNAs in non-invasive diagnostics and their prognostic potential as biomarkers are presented.

## 1. Introduction

B-cell precursor acute lymphoblastic leukemia (BCP-ALL) is a frequent hematologic neoplasm, characterized by an uncontrollable growth of immature B lymphocytes. It is one of the most common leukemia types in children. It is responsible for a large proportion of childhood cases of acute lymphoblastic leukemia (ALL) in the age group of 2 to 5 years, evaluated to be more than 80% of cases. Nearly 90% of pediatric patients who suffered from BCP-ALL are cured, but still, it is considered to be one of the main causes of death due to cancer among pediatric patients [1]. Childhood BCP-ALL is characterized by the heterogeneous occurrence of genetic abnormalities and the most common genetic aberrations, which are responsible for 70 to 75% of cases of this disorder, including *ETV6*::*RUNX1*, hyperdiploidy, hypodiploidy, *TCF3*::*PBX1*, *BCR*::*ABL1*, and *KMT2A* subtypes. The recognition of a concrete subtype is important because of factors such as risk estimation and specification of therapy [2]. MicroRNAs (miRNAs) can act as important factors in the diagnosis and prognosis of childhood BCP-ALL. These molecules are inextricably linked with the processes present in the progress of each BCP-ALL subtype. Therefore, determining their stability in circulation points to the potential utility of miRNAs as biomarkers in the development of BCP-ALL [3]. In this review, we describe the possibilities of diagnostics and prognostics of BCP-ALL, with the use of selected miRNAs.

## 2. Background

### 2.1. Non-Coding RNAs

The minority (less than 2%) of the human genome comprises protein-coding genes, which were the focus of research by scientists for decades. Until recently, the remaining, non-coding part (about 98%) of the genome was considered non-functional “junk”. However, the reports of the ENCODE project launched in 2005 are contradictory to the tenet that non-protein-coding part is useless. The ENCODE effort showed that about 80% of our genome has the ability to copy into non-coding RNAs (ncRNAs) [4,5]. This type of molecule belongs to functional RNAs, and these participate in both physiological and pathological biological processes, and even in the development of oncological diseases [6,7]. ncRNAs are a wide group of molecules of different sizes and regulatory capacities. There are two main groups of ncRNAs named housekeeping ncRNAs and regulatory ncRNAs that differ in regulatory function. Based on their size, ncRNAs are classified as long ncRNAs (lncRNAs) including molecules greater than 200 nucleotides and small ncRNAs (sncRNAs) including RNAs such as tRNA-derived small RNAs (tsRNAs), PIWI-interacting RNAs (piRNAs), or miRNAs [4,5,7].

In many types of cells, at different stages of their development, lncRNA expression is observed. This type of molecule has significant effects on metabolism, differentiation, and the cell cycle, so it may contribute to the development of cancers, including childhood BCP-ALL. lncRNAs have the ability of, inter alia, chromatin and DNA modification as well as RNA transcription or mRNA degradation [8,9].

tsRNAs arise from precursor or mature tRNA sequences by the action of nuclease. They are 14-40 nucleotides long and can influence cellular processes, including gene regulation, gene silencing, and reverse transcription. These molecules can interact with various proteins to influence the regulation of gene expression at the pre-transcriptional and post-transcriptional levels [10,11].

piRNAs are molecules of about 23-32 nucleotides in length and are derived from single-stranded precursors including a piRNA cluster. These molecules are present in both embryonic and somatic cells and are able to form a complex with PIWI proteins, which affects the oncogenesis process by participating in genome rearrangement or epigenetic regulation [12,13].

### 2.2. MicroRNAs

The non-protein-coding part of the human genome is of interest to scientists, and there are many studies about its role and usage in diagnostic–therapeutic processes. One of these molecules is miRNA, the small (about 21–23 nucleotides in length) single-stranded type of ncRNA [14]. In 1993, Ambros and Ruvkun discovered the first miRNA, Lin-4, in Caenorhabditis elegans. In the same species of nematode, the second miRNA, named let-7b, was discovered in 2000 by Reinhart et al. That finding was the turning point in studies about miRNA, and since then, thousands of these molecules have been observed in many species, including humans [14,15,16].

#### 2.2.1. Biogenesis of microRNAs

The biogenesis of miRNA is a multi-stage process located in the nucleus and cytoplasm, respectively. miRNA molecules have various genomic origins such as coding genes, intron regions, and gene promoter regions [14,17,18]. However, only a minority of the miRNA molecules reside in introns. That fraction of miRNA is called mirtrons and originates from non-canonical pathways in contrast to the intergenic miRNA, which comes through the canonical pathways [14,19,20]. The process of biogenesis starts with the transcription of a long primary miRNA (pri-miRNA) and can be done depending on the host genes or independently (especially for mirtrons) [14,20,21]. A 7-methylguanosine cap, a 3′ poly(A) tail, and one or more precursor miRNA (pre-miRNA) hairpins are all components of a pri-miRNA [20]. There are two types of RNA polymerase that take part in the creation of pri-miRNA in the nucleus. One of these enzymes is RNA Pol II, the preferred polymerase for this transcription process. Specific features of pri-miRNA such as length and sequences of uridine residues and the fact that characteristics of promoters are typical of RNA Pol II are responsible for the preference of this polymerase. The transcript of exceptional kinds of miRNAs (e.g., miRNA 142) occurs with the second type of enzyme called RNA Pol III [14].

In the canonical pathway of miRNA biogenesis, the pri-miRNAs generated in the transcription are cleaved into pre-miRNA characterized by a stem-loop structure and 60-70 nucleotides in length. This stage occurs in the nucleus through a microprocessor complex composed of one RNase III DROSHA and two double-stranded RNA binding protein (RBP) DiGeorge Syndrome Critical Region 8 (DGCR8, also known as Pasha) molecules [19,22]. A major role of the DROSHA is cleaving the 5′ cap and polyA in the pri-miRNA [18]. Furthermore, the DROSHA measures 11 base pair distance from the basal single-stranded RNA–double-stranded RNA junction and cleaves the hairpin [22]. DGCR8, a co-factor of the DROSHA, is involved in the process of pre-miRNA formation. The functions of DGCR8 include recognizing and stabilizing RNase to the apex of the hairpin through the double-stranded RNA binding and C′-terminal domains [20,23]. The pre-miRNA formed as a result of the above-described processes is then transported into the cytoplasm through the nuclear membrane and the nuclear pore complex. This step occurs with the protein Exportin-5 (EXP-5) and the cofactor Ran-GTP. Furthermore, the EXP-5 plays an additional role in protecting pre-miRNA from degradation. These two molecular elements create a complex with pre-miRNA and after its transportation release it into the cytoplasm [18,20].

The cytoplasmic stage of miRNA formation, in the canonical pathway, leads to miRNA duplex creation from pre-RNA. The pre-RNA molecules are modified into these duplexes through the second RNase III known as DICER. Protein activator of protein kinase R (PACT) and TAR mRNA-binding protein (TRBP) are the cofactors of the DICER enzyme; thus, they are necessary for this process [17,24]. Under the influence of the TRBP, the DICER can change the precursor cleavage position and guide strand selection. Using the capacity to modify this location, TRBP with PACT contributes to creating miRNAs of various lengths.

The structure of DICER is composed of many domains and has a shape similar to the letter L. RNase IIIa, RNase IIIb, N-terminal helicase, C-terminal double-stranded RNA-binding domain (dsRBD), Platform-Piwi/Ago/Zwille (PAZ)-connector helix domain, and DUF283 are the elements creating DICER [21,25,26]. Each part of this enzyme has a different function in pre-RNA transformation, and the most important element is described below. The dimer formed by RNase IIIa and RNase IIIb is the catalytic core of DICER and is generally responsible for cutting pre-miRNA strands and releasing the duplexes of miRNA [21,26].

**Figure 1:**
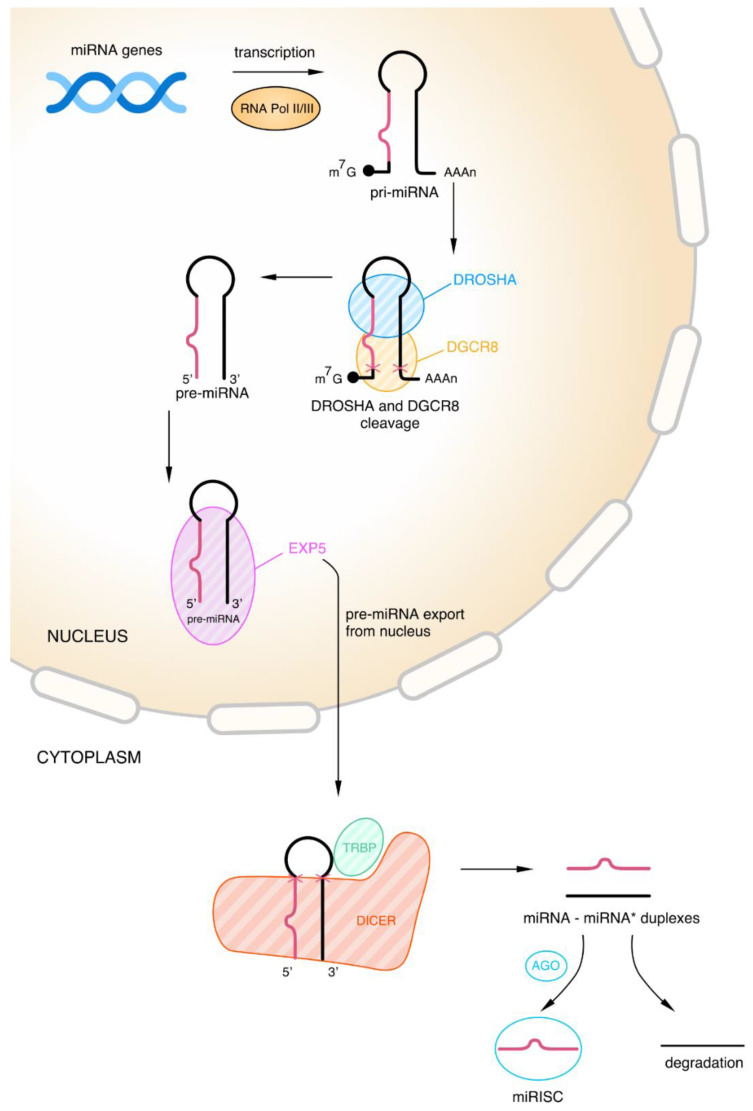
Canonical pathway of miRNA biogenesis. As a result of transcription of miRNA genes by RNA Polymerase II (Pol II) or, in a few cases, Polymerase III (Pol III), the primary miRNA (pri-miRNA) is produced. Then DROSHA and DiGeorge Syndrome Critical Region 8 (DGCR8) take part in cleaving the pri-miRNA; i.e., they cleave the 5’ cap and polyA tail (the red crosses symbolize the place of the cut). As a result of this process, a precursor miRNA (pre-miRNA) is formed, which leaves the cell nucleus with the help of the protein Exportin-5 (EXP-5). In the cytoplasm, the pre-miRNA is further cleaved by DICER and TAR mRNA-binding protein (TRBP) (the red crosses symbolize the place of the cut). This process produces miRNA–miRNA duplexes *. One part of the duplex is degraded and the other part, in the presence of Argonaute protein (AGO), forms the polyA-specific ribonuclease (miRISC) complex.

The result of the cleaving process with DICER is miRNA–miRNA* duplex formation, where miRNA* is the passenger strand and miRNA is the guide strand. Later, the miRNA* strand is degraded and the second one is loaded into Ago protein and forms the core of the miRNA-induced silencing complex (miRISC). Mature miRNA formed with the participation of both arms of the duplex plays the role of gene expression regulator, which can be transported back to the nucleus through nuclear pores [18,27,28]. The main roles of the miRISC are recognition and silencing of the targeted transcript, but the protein composition outside its core has an influence on the functioning and kinetics of miRISC as well [29]. Targets of this complex are recognized by miRNAs taking advantage of complementarity at nucleotides 2–8 of the 5′ end, and thus the recognition of sequences localized in 3′ untranslated region (UTR) by the miRISC leads to the repression of the translation process and/or to the strengthening of the degradation of the transcript [17]. The canonical pathway of miRNA biogenesis, in the steps, is shown in Figure 1.

Apart from miRNA molecules formed in a canonical pathway, non-canonical miRNAs also play an important role in the pathogenesis of neoplastic diseases. The biogenesis of these molecules is different and takes place independently of DROSHA or DICER [30,31].

The main group that bypasses the DROSHA cleavage is mirtrons, the pre-miRNAs originating from introns. These molecules are small and do not have the one helical turn needed for DROSHA cleavage; therefore, instead of this cut, mirtrons go through the lariat-debranching process performed by debranching enzyme 1 (DBR1). After that, they can be transported, cooperating with EXP-5, straight to the cytoplasm, and there they can be modified by DICER like pre-miRNA in the canonical pathway [20,31,32].

There is evidence that some miRNAs are derived from different non-coding molecules such as small nucleolar RNA (snoRNA) and transfer RNA (tRNA). snoRNA-derived miRNAs are similar in length to the canonical RNAs of approximately 21 nucleotides. The correct tRNA folding plays an important role in the formation of tRNA-derived miRNAs. The presence of the RNA chaperone La is essential to avoid tRNA misfolding because the lack of it causes pre-tRNA to become a substrate for DICER and the miRNA to be generated [31,32].

In addition to the production of miRNAs from non-coding molecules, there is one more way of their biogenesis independent of DROSHA, which uses transcription using Pol II. The release of the substrate hairpins for the DICER enzyme is caused by the early termination of gene transcription by Pol II. In the canonical pathway, the pri-miRNA cap is removed by the DICER enzyme, in contrast to the cap of these transcribed products, which affects nuclear export with Exportin-1 (EXP-1) [32].

**Figure 2:**
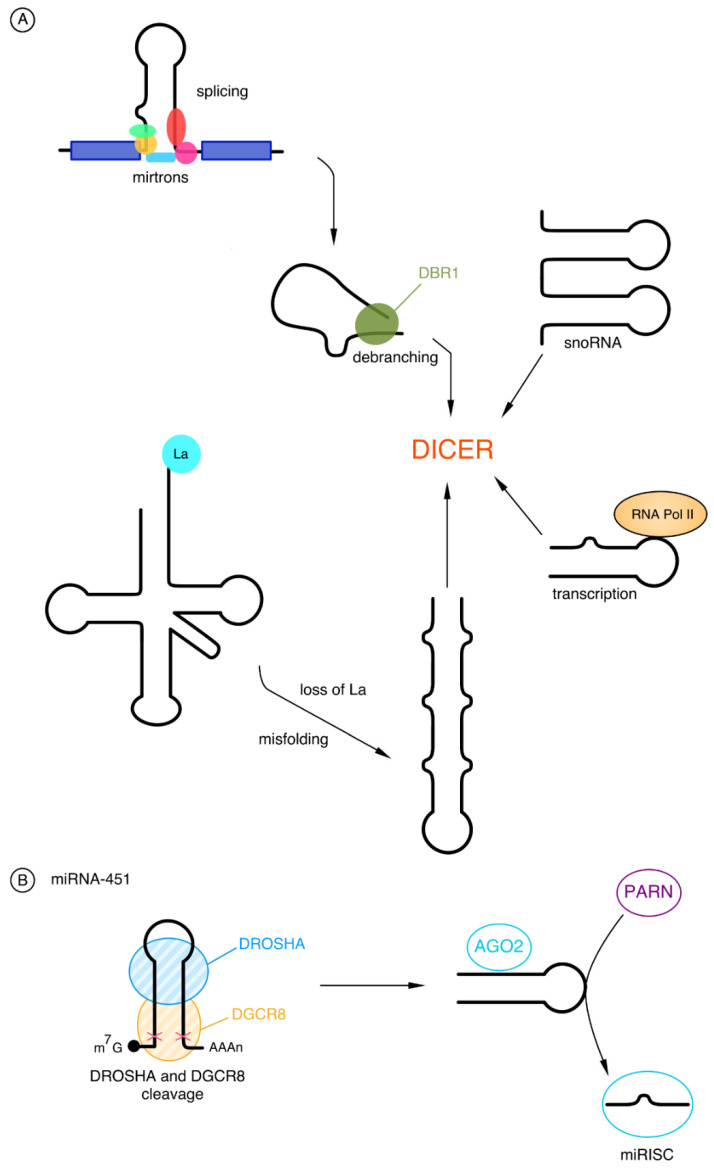
Non-canonical pathway of miRNA biogenesis: (**A**) The pathway independent of DROSHA. The first group of DROSHA-independent cleavage is mirtrons. They are generated by splicing and then by debranching enzyme 1 (DBR1) performing the lariat-debranching process. The next group is snoRNAs which are recognized by DICER. The third one is products of transcription occurring with the use of RNA Polymerase II (Pol II), which are then used by DICER. The last example is the tRNA. The misfolding resulting from the loss of RNA chaperone La leads to a form that can be used by DICER. (**B**) The pathway independent of DICER—miRNA-451. One example of a DROSHA-independent molecule is miRNA-451. Following the cleaving process involving DROSHA and DiGeorge Syndrome Critical Region 8 (DGCR8), the molecule is loaded into the Argonaute protein 2 (AGO2). This is followed by the treatment by polyA-specific ribonuclease (PARN) and the formation of polyA-specific ribonuclease (miRISC).

miRNAs produced in a mechanism independent of DICER are extremely rare; thus, so far, only one molecule has been described—miRNA-451. The core of miRNA-451 is about 17 base pairs, which is why DICER does not recognize them, because of their short length. Thus, when cleaved by DROSHA, pre-miRNA-451 bypasses DICER and is loaded into the AGO2 protein. AGO2 trims pre-miRNA-451 in the middle of the 3’ end and is then further cleaved by polyA-specific ribonuclease (PARN) [32,33,34]. The non-canonical pathway of the biogenesis of miRNAs is shown in Figure 2.

#### 2.2.2. Gene Regulation by microRNAs

One of the roles of miRNAs is the regulation of gene expression, which occurs through various mechanisms influencing post-transcription and transcription processes. These mechanisms include transcriptional gene silencing (TGS), transcriptional gene activation (TGA), and post-transcriptional gene silencing (PTGS) using RISC, which are described in this part of the chapter [35,36].

The regulation of genes by miRNAs mainly takes place at the post-transcriptional level. At this stage, the miRNA acts on gene expression through RISC. The process occurs by recognizing the miRNA and then binding them by a sequence present on the mRNA. This fragment of mRNA is called the miRNA response element (MRE) and usually is placed at 3′UTR mRNA, but it can be located at 5′UTR or in the sequences that code proteins [15,36]. Further proceeding depends on the complementarity of the MRE and miRNA. Incomplete complementarity occurs in most cases, leading to inhibition of RISC-mediated translation and disintegration of targeted mRNA. However, when the miRNA and MRE match perfectly, the endonuclease AGO2 is activated and then mRNA is cleaved [15].

miRNAs may also affect gene recruitment by inducing TGS. Stabilization of repressors or recruitment of corepressors, caused by the binding of the miRNA to the RNA promoter or to a site in the promoter region, contributes to silencing the transcription of the target gene. Nuclear miRNAs are presumed to be able to regulate the biogenesis of other miRNAs by interacting with relevant pri-miRNAs [35].

Regulation of genes by miRNAs can be mediated by induction of TGA, which can occur in several ways. Examples of this type of regulation include the binding of miRNAs to enhancers or the process of bi-directional transcription of the human genome. Recruitment of the protein complex with transcriptional activators to the promoter gene is another mechanism used for the induction of TGA, but there is one more way based on the creation of the positive feedback loop on transcription [35].

## 3. miRNAs in Childhood BCP-ALL

### 3.1. Downregulated miRNAs

As previously mentioned, miRNAs may perform as biomarkers in the diagnosis and prognosis of childhood BCP-ALL. Some of them can be differentially expressed among BCP-ALL patients. The underexpression of certain miRNA types is crucial for the development of BCP-ALL, and thus these molecules should serve as biomarkers. They are noninvasive and simple to exhibit in a specimen from a patient, and because of that, miRNAs are gaining the attention of researchers. In this section, selected downregulated miRNAs are presented [37].

#### miRNA-200c and miRNA-326

The miRNA-200 family is a group of functional miRNAs that includes miRNA-141, miRNA-200a, 200b, 200c, and miRNA-429. These five miRNAs are situated in two clusters of chromosomal location: the miRNA-200ba/429 cluster on chromosome 1p36 and the miRNA-200c/141 cluster on chromosome 12p13. The miRNA-200 family may be categorized into two sequence clusters according to the seed sequences, which determine the direct binding target of miRNA. The seed sequence of the miRNA-200bc/429 cluster (miRNA-200b, 200c and 429) is AAUACUG, while that of the miRNA-200a/141 cluster (miRNA-200a and miR-141) is AACACUG with one nucleotide difference [38]. This group functions as a master mesenchymal–epithelial transition regulator controlling the expression of epithelial marker E-cadherin, which is a cell–cell adhesion molecule, transmembrane glycoprotein 120-kDa. This glycoprotein performs in trophoblastic differentiation. Moreover, cellular adhesiveness can be crucial to the possibility of epithelial tumor cells invading and metastasizing [39,40]. Of all the miRNA molecules of the miRNA-200 family, the miRNA200c is the most interesting factor in the development of pediatric BCP-ALL.

According to recent discoveries, miRNA-200c acts as an inhibitor on metastases. The analysis comprised 18 studies based on 3676 samples from patients with various neoplasms. miRNA-200c was tested in eight of these studies. The results suggest that miRNA-200c downregulation has a diagnostic value in the diagnostics of malignancies. In addition, the examination of dysregulated expression of miRNA-200c in many neoplasms has been conducted. The stability of miRNA-200c in blood serum is yet another important factor in determining its role as a potential biomarker [41]. Along with the miRNA-200c, miRNA-326 has been identified as a possible biomarker in BCP-ALL. miRNA-326 is included in the miRNA-15/107 gene group, and its precursor is situated within chromosome 11 in intron 1 of the beta-arrestin gene (ARRB1). This molecule is examined as an immune-related miRNA because of its influence on the mediation of immune response [42].

miRNA-326 takes part in various cellular processes. It has been proved that miRNA-326 takes part in metastasis, invasion, proliferation, and apoptosis within the tumor cells in several cancers. It acts as an inhibitor in invasion and metastasis; thus, its level of expression is downregulated in some neoplasm cells. This molecule is also present in a couple of signaling pathways, such as the Hedgehog/Gli (Hh/Gli) signaling pathway [43]. miRNA-326 has been reported to play a role in response to chemotherapy by increasing the risk of chemoresistance with the association of multidrug resistance (MDR). The research of Elaheh S. Ghodousi and Soheila Rahgozar has shown that downregulation of miRNA-326 is inversely correlated with overexpression of ABCA2 and ABCA3 in minimal residual disease-positive (MRD+) patients, which is why it might be a prognostic factor for drug resistance in BCP-ALL. The investigation referred to 46 bone marrow samples inclusive of 36 BCP-ALL cases. The expression profile in MRD+ ALLs was compared with an MRD- group, and patients with relapsed ALL were also contrasted with MRD- ALLs. Importantly reduced expression of miRNA-326 was noted in MRD+ and relapsed patients in comparison with MRD- patients, which confirms the prognostic value of miRNA-326 in pediatric BCP-ALL [44].

Certain miRNAs affect concrete genes. Examples include miRNA-326 and miRNA-200c. These molecules work on transporter genes ABCA-3 and ABCA-2, and both show a degression in level throughout the BCP-ALL patients. The outcome presented a downregulation of miRNA-326 and miRNA-200c expression in BCP-ALL patients compared to healthy individuals. The abnormalities in the expression level of miRNA-326 and miRNA-200c demonstrated that both molecules have a potential role in the BCP-ALL diagnostic process. These changes discovered in leukemic patients may serve as prognostic factors for this malignancy. Thus, these molecules may perform as diagnostic biomarkers in BCP-ALL development [3].

### 3.2. Down- and Upregulated miRNAs

Some miRNA molecules show different expression levels depending on the samples or genetic variants of ALL. The following are molecules that are characterized by under- or overexpression depending on the factors mentioned above.

#### 3.2.1. miRNA-100

It is known that miRNA-100, which is located on chromosome 11q24.1, is involved in inhibiting cellular proliferation, so it is possible that it also affects malignant lymphopoiesis [45,46,47]. In patients with ALL, miRNA-100 has been found to be responsible for an increase in dexamethasone-induced cellular apoptosis [45,48,49]. Thus, the occurrence of miRNA molecules may be related to the number of white blood cells. Studies conducted by Li et al. and Oliveira et al. found that children who had low miRNA-100 expression before starting cancer therapy had a white blood cell (WBC) level of above 50 × 10^9^ cells/L [49,50].

In the study by Hassan et al., there was a negative correlation between miRNA-100 levels and the incidence of ALL. The study group consisted of 85 children with leukemia, 50 of whom had the B-cell subtype, and the median blast count of all patients was 91% [45]. It is important to emphasize that the samples used in the study were from bone marrow (aspirate in the case of Hassan’s research, not specified in the studies of Li and Oliviera), as the study by Xue et al. achieved different results from those described above. The underlying cause for these differences is likely a discrepancy in the study material. The second study found significant overexpression of miRNA-100 in children with ALL, but the study material was plasma samples [45,48]. Concordant results were also obtained by Xue et al. and by Swellam et al. [46].

In addition to the first study, a correlation was detected between low miRNA-100 expression and shorter DFS. In children with ALL, those with high miRNA-100 expression had a mean OS of 36.18 months, while those with low expression of this molecule had an OS of 53.56 months [45]. Despite the detection of a link between the above data, there have been earlier reports that miRNA-100 levels have no effect on DFS (Oliviera et al.) or that low expression determines shorter OS and DFS (Li et al.) [45,49,51].

There are various genetic variants in BCP-ALL that indirectly influence the course of the disease. Xue et al. investigated the association of the occurrence of some with a cellular expression of miRNA-100. The study included 831 children with ALL, 728 of whom were characterized by the B-cell subtype. Among the different genotypes, the rs543412 homozygous TT variant, compared to the CC variant, had a significantly greater protective role and lower miRNA-100 expression in patients [48]. In addition, Schotte et al. and Oliveira et al. showed that higher miRNA-100 expression occurs in children with BCP-ALL with associated t(12;21) and no hyperdiploid karyotype. Low levels of this molecule, in turn, correlated with cases of leukemia without translocation, associated with CD34+ cells [50,52,53].

#### 3.2.2. miRNA-181a

miRNA-181a has been shown to be upregulated in most pediatric ALL subtypes compared to controls [54,55,56]. The exception is the BCP-ALL subtype associated with ETV6/RUNX1, which is downregulated for this particular miRNA molecule. It may act as an oncomiR or as a suppressor molecule depending on the context [57,58].

Information about miRNA-181a subtypes in the human genome is encoded on the miR181a1/b1 cluster on chromosome 1 and the miR181a2/b2 cluster on chromosome 9. The site of preferential expression of miRNA-181a is precursor B cells. Therefore, under the ectopic expression of this molecule, hematopoietic progenitors differentiated toward the more mature B-cell phenotype [59,60].

Several genetic variants of BCP-ALL may be associated with a better prognosis, one of which is t(12;21)(p13;q22), which is also the most common in this type of leukemia. This translocation results in the *ETV6::RUNX1* fusion gene, which contains a putative miRNA-181a binding site [61,62]. Schotte et al. discovered that in patients representing this translation, the expression of miR181a-1 was 5-fold lower than that in children with other ALL subtypes [52]. Further experiments examining the relationship between *ETV6::RUNX1* and this particular miRNA molecule suggested that they may regulate each other. Using this coregulation, miRNA-181a was targeted to the fusion protein, resulting in a downregulation of the oncoprotein [62,63]. Moreover, after delivery of the ectopic miRNA, inhibition of CD10 hyperexpression was observed in primary samples of *ETV6::RUNX1*-positive patients [63].

An increase in the level of miRNA-181a is associated with a milder course of the disease and a lower risk of recurrence [62]. Nabhan et al. compared miRNA-181a levels in serum samples from 30 pediatric ALL patients (77% pre-B immunophenotype) with those from 30 healthy children, and it has been shown that miRNA-181a is significantly downregulated in ALL patients [64]. Additionally, their research proved that miRNA-181a in combination with its target protein Smad7 and TGF-β1 together have high accuracy (96.7%) in detecting pediatric ALL. The antileukemic effect that miRNA-181a induces by targeting *ETV6::RUNX1* can be used in studies on further forms of BCP-ALL therapy [62,64].

### 3.3. Upregulated miRNAs

As indicated above, downregulated miRNAs have a significant impact on the course and prognosis of BCP-ALL. In addition, overexpression of certain miRNAs can be observed in this disease.

#### 3.3.1. miRNA-155

Many pathways of molecular and gene expression are involved in BCP-ALL, one of which is the overexpression of miRNA-155. Studies have shown that miRNA-155 has many functions. It may act as an oncogene or anti-oncogene and its role depends on the type of target cell [65,66,67]. The miRNA-155 molecule is considered to play an important role in the development and modification of cells from the hematopoietic lineage, especially since it was found as a gene in the integrin cluster of B cells in chickens. Later, MIR155 (MIR155HG), a host-coded gene formerly known as B-cell integration cluster (BIC), was identified in humans. Its location on the chromosome is 21q21, and the seed sequence of miRNA-155 is UAAUGCU [65,68,69]. High expression of miRNA-155 is characterized by B cells, T cells, and macrophages, among others. At the same time, this molecule is involved in cellular immune responses and the hyperproliferation of leukemic blasts [69].

Due to the potential involvement of miRNA-155 molecules in many immune mechanisms, their impact on the diagnostic and therapeutic process of acute leukemia in children has been considered. A study by Liang et al. showed that miRNA-155 may have prognostic value in ALL patients. The study group consisted of 42 children, 41 of whom were characterized by B-cell lineage leukemia. Bone marrow samples were collected from the patients and subjected to qRT-PCR analysis, which showed that miRNA-155 levels were significantly higher in children who relapsed (10 patients with B-ALL) and those with central nervous system involvement. The presence of central nervous system leukemia also correlated with shorter overall and event-free survival [70]. In patients with elevated miRNA-155 expression, the increased cellular proliferation of all four ALL cell lines and decreased apoptosis rate were observed, which decreased and increased sequentially after the administration of inhibitors of this molecule and became comparable to those of the control group. Based on these results, it can be concluded that miRNA-155 is involved in the promotion of proliferation and inhibition of apoptosis in ALL, and it may be a potential therapeutic target [70].

Many studies have searched for the target protein miRNA-155 via protein suppression effects. In this process, SH2-domain contains inositol-5′-phosphatase 1 (SHIP1), which is high in bone marrow cells and B-cells, was discovered. SHIP1 regulates proliferation and differentiation and is a negative regulator of the PI3K/AKT signaling pathway; its underexpression may result in Act activation. In studies, SHIP1-deficient mice developed myeloproliferative disease and leukemia. miRNA-155 binds to the 3′UTR of SHIP1 mRNA, thereby inhibiting its translation. Therefore, upregulation of miRNA-155 results in the suppression of SHIP1 expression, and this may lead to the development of human MPD and myeloid leukemia [71,72].

In tumors of the nervous system, such as glioblastoma multiforme [73], *ZNF238* has been found to be responsible for tumor-suppressive activity. In the above study by Liang et al., a correlation was observed between miRNA-155 and *ZNF238*, which may presumably be associated with cell proliferation in BCP-ALL. It was noted that ZNF238 expression was inhibited in the cells infected by miRNA-155 at the protein and mRNA levels. This inhibition leads to an increase in ALL cell proliferation [70].

In a study conducted by Akpinar et al., higher levels of miRNA-155 were observed in B-ALL patients compared to healthy patients. The results were as follows: the median in healthy subjects was 28.5 (range 25.96–33.71) and the median in leukemia patients was 127.47 (range 0–46,148.62). The research included a study group of 21 leukemia patients among whom there were 12 children with BCP-ALL [74]. Similar observations were noted by El-Khazragy et al. who demonstrated a significantly higher expression of miRNA-155 among 21 children with BCP-ALL. In addition, it was noted that high levels of this molecule also correlate with a high percentage of blasts (above 25%), a total leukocyte count (above 50 × 10^9^ cells/L), a high relapse rate, and lower levels of hemoglobin (≤60 g/L). All of the above-mentioned features demonstrate the probable correlation of miRNA-155 with a poor response to the treatment of leukemia [75]. A meta-analysis by Zhang et al. showed that miRNA-155 expression is associated with lower overall survival and is significant in high-risk B-ALL [53,68,75].

miRNA-155 may also have potential in controlling the course of BCP-ALL therapy. A study by Duyu et al. on 34 patients with B-ALL showed that after 6 months of anticancer therapy, miRNA-155 values decreased significantly compared to values before treatment [53,74,76]. Complete remission is also associated with very low miRNA-155 expression, as observed in the study by Hassan et al. [77]. El-Khazragy et al. also showed that miRNA-155 levels decreased 10-fold from a baseline level after treatment [75].

**Figure 3:**
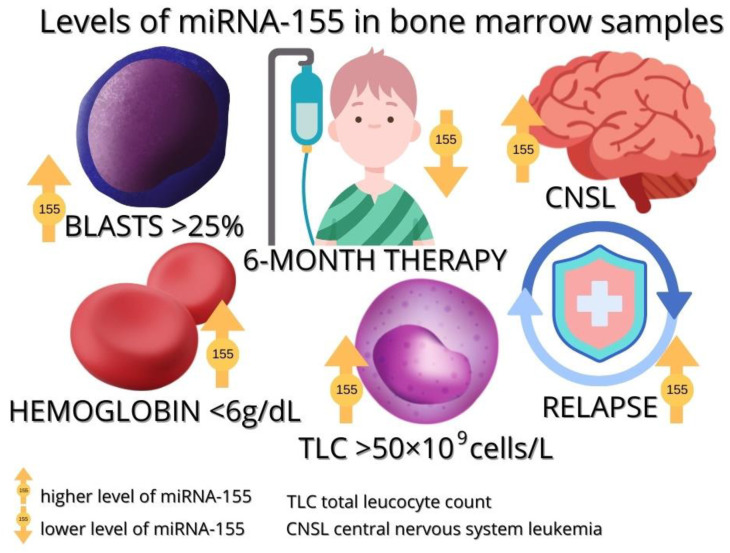
The level of miRNA-155 in bone marrow samples from children with ALL.

Figure 3 summarizes the levels of miRNA-155 in pediatric bone marrow samples according to the characteristics associated with the disease.

#### 3.3.2. miRNA-125b

One of the ncRNA molecules found to be highly expressed in BCP-ALL is miRNA-125b [53,78]. It has the ability to induce neoplastic transformation in the cells of both lymphoid and myeloid lines. Additionally, this particular miRNA plays a key role in several regulatory networks such as the Wnt, PI3K/Akt, STAT-3, MAPK, NF-κB, and p53 pathways [79]. Mature miR-125b is from two loci located on chromosomes 11q23, *hsa-miR-125b-1* for miRNA-125b-1, and 21q21, *hsa-miR-125b-2* for miRNA-125b-2. The origin of miRNA-125b-1 is *MIR100HG*, affiliated with the lncRNA class. In addition to miRNA-125b-1, *MIR100HG* is the cluster host gene for miRNA-100 and let-7a-2. miRNA-125b-2 is derived from a common miRNA cluster with miRNA-99a and let-7c [80].

Multipotent bone marrow progenitor cells and myeloid cells are the sites of expression of miRNA-125b, while in B cells it is silenced in healthy individuals. The dysregulated expression of this molecule results in impaired transport and enhanced proliferation of immature B cells, as well as their stunted maturation [81].

miRNA-125b is upregulated in BCP-ALL patients carrying the t(11;14) translocation (q24;q32) involving the immunoglobulin heavy chain gene (*IGH*) locus [55]. Chapiro et al. showed that the level of miRNA-125b was significantly higher in samples from patients with translocation than in those without it [82]. In these BCP-ALL patients, this particular miRNA molecule targets ARID3a, inhibiting caspase activation through a mechanism that is independent of p53 and BAK1, resulting in the evasion of apoptosis [83,84]. The overexpression of the *hsa-miR-125* cluster was also observed to be specific for leukemic cells in patients with BCP-ALL associated with the *ETV6*::*RUNX1* mutation [85]. qRT-PCR analysis revealed that miRNA-125b is also involved in the pathogenesis of BCR-ABL-associated BCP-ALL. In patients with BCR-ABL translocation, researchers observed a strong upregulation of miRNA-125b with reduced p53 expression and increased survivin expression [86]. This specific miRNA molecule may mediate leukemia pathogenesis by accelerating the carcinogenicity of the BCR-ABL fusion protein [83]. Another subtype of BCP-ALL that has been reported to have high miRNA expression from the miRNA-125b cluster is ERG-related leukemia [78].

The prognostic value of miRNA-125b in ALL therapy was proven in a study by Piatopoulou et al. Decreased miRNA-125b levels on the day of diagnosis and elevated miRNA-125b levels on day 33 of chemotherapy were associated with treatment resistance and shorter overall survival with the Berlin–Frankfurt–Munster (BFM) backbone protocol [87]. Another study linked miRNA-125b upregulation to daunorubicin and vincristine resistance in the treatment of TEL-AML1-positive ALL [52].

The participation of miRNA-125b in many signaling pathways and cancer processes points to several potential clinical applications of this molecule. It can be tested for use as a diagnostic and prognostic biomarker, as well as for being a therapeutic target [79,87].

#### 3.3.3. miRNA-146a

The miRNA-146a molecule, by targeting tumor suppressors and oncogenes, may promote increased proliferation, abnormal cell growth, and reduced cell death. The effect of miRNA-146a upregulation on genes such as N-RAS, RAS, AMPK-alpha, PBX2, ErbB4, TRAF6, LIN28, and NUMB may lead to the onset of ALL [88]. The chromosomal location of hsa-mir-146a is 5q33.3 [89]. Low levels of miRNA-146a are synthesized in hematopoietic progenitor cells. However, miRNA expression may be upregulated by a viral or fungal infection [90,91]. High levels are constitutively expressed by Ly-6Clo monocytes and epidermal Langerhans cells [92,93,94]. In addition, miR-146a has a proven effect on the maturation of B lymphocytes. [95,96].

It seems that upregulation is common in the ALLs of children. The only exception to this is the *TCF3*-r BCP-ALL subtype [52,97]. In a study by Wang et al., miRNA-146a expression was significantly higher in acute leukemia patients compared to patients in the control group. Higher levels of this particular molecule were present in patients with ALL. Furthermore, miRNA-146a has been shown to have a leukogenic effect by downregulating CNTFR and activating the JAK2/STAT3 pathway in Jurkat and HL-60 cells. [95]. In ALL patients overexpressing miRNA-146a, survival is poorer, but this is a common feature for both ALL and AML patients [98].

The prognostic role of miRNA-146a was validated by examining its expression in 42 children and 24 adults with ALL and comparing them to controls. High levels of the described miRNA before chemotherapy have been shown to decrease after treatment [88]. It also seems that miRNA-146a may contribute to the therapy of ALL, enhancing the effect of prednisolone on treatment [99].

Taking into account the relationship between the level of miRNA-146a and the prognosis, it may be a helpful parameter not only in BCP-ALL but also in T-ALL therapy. 

A comparison of downregulated and upregulated miRNAs is shown in Table 1.

## 4. Conclusions

Non-coding molecules such as miRNAs, derived from different regions of genes, influence the development of pathological processes leading to the development of oncological hematological diseases, including childhood BCP-ALL. Abnormalities in the expression of miRNA molecules, lead to the disorder of the proper functioning of the stem cells of the lymphoid lineage, which makes them good material for diagnostics and prognosis. The facility of isolation and the availability of samples for miRNA studies create new possibilities in monitoring patients with BCP-ALL and give a chance to react quickly and determine prognosis or response to treatment. The miRNA molecules described above may potentially have a prognostic and diagnostic value in the BCP-ALL neoplastic process, although there are no reports on their current use in clinical practice.

## Figures and Tables

**Table 1:** The characteristics of selected miRNAs in childhood BCP-ALL.

Locus on Chromosome	microRNA	Expression in BCP-ALL	Target of miRNA	Function mRNA of miRNA/Target	Ref.
12p13	miRNA-200c	downregulation	*ABCA2*, *ABCA3* *EMT/MET*	inhibition of tumor metastases, involved in leukemogenesis (?) multidrug resistance	[3,38,42,44,100]
11q1	miRNA-326	downregulation	*ABCA2*, *ABCA3*	inhibition of invasion and metastasis, multidrug resistance chemoresistance	[3,43,44,101]
1q32.1 * 9q33.3 **	miRNA-181a	downregulation	*Smad7* ↑ *ETV6::RUNX1*	increase in proliferation and decrease in apoptosis	[59,60]
		upregulation	*TLR4, TLR8* *IRF8, IL6R*	inhibition of innate immunity and inflammation	[3]
11q24.1	miRNA-100	downregulation	*FKBP51* ↑ the antiapoptotic gene *MCL1* ↑	regulation of cell proliferation and dexamethasone-induced apoptosis	[45,46,47,102]
		upregulation	*IGF1R/mTOR* (*MCL1* ↓)	cell proliferation, differentiation, and survival stimulation; rapamycin-mediated enhancement of dexamethasone-induced apoptosis	
21q21.3	miRNA-155	upregulation	*SHIP1* *Mxd1/Mad1* (*BCL6*↓) *HDAC4* *SMAD5*	promotes B cell proliferation, carcinogen, inhibits the proliferation of human hematopoietic progenitor cells by signaling TGF-β pathway, chemoresistance	[3,66,67,103]
21q21.1	miRNA-125b	upregulation	*PPP1CA*, *BTG2*, *PTEN* ↓ *P53*, *Bak1*, *Bmf transcript* ↓	increase in proliferation and decrease in apoptosis, oncogene	[3]
5q33.3	miRNA-146a	upregulation	*N-RAS*, *RAS*, *AMPK-alpha*, *PBX2*, *ErbB4*, *TRAF6*, *LIN28*, *NUMB*, *CNTFR*	increased proliferation, influenced maturation of B lymphocytes, inhibited apoptosis	[85,88,89]

* miRNA-181a1 (cluster with miRNA-181b1). ** miRNA-181a2 (cluster with miRNA-181b2).
